# Radiological Diagnosis of Axillary Artery and Brachial Plexus Involvement in a Proximal Humerus Fracture-Dislocation: A Case Report

**DOI:** 10.7759/cureus.92425

**Published:** 2025-09-16

**Authors:** Nadir Parkar, Tanujan Thangarajah

**Affiliations:** 1 Department of Orthopaedics, University College London Hospitals, London, GBR

**Keywords:** brachial plexopathy, ct, ct angiogram, proximal humerus fracture, proximal humerus fracture-dislocation

## Abstract

Proximal humerus fracture-dislocations are uncommon in elderly patients and rarely associated with neurovascular compromise. The anatomical proximity of the axillary artery and brachial plexus to the humeral head places them at risk during trauma, but diagnosis can be challenging when distal pulses are preserved. We present the case of a 79-year-old female who sustained a fracture-dislocation of the proximal humerus following a low-energy fall. CT angiography demonstrated humeral head displacement into the axilla with bony impingement on the axillary artery compression and brachial plexus tenting without transection. The patient underwent reverse total shoulder arthroplasty with neurovascular exploration. The patient developed postoperative neuropraxia, which gradually improved with rehabilitation. This case highlights the diagnostic challenge of vascular injury without overt clinical signs and underscores the role of CT angiography in surgical planning. Early imaging allowed a multidisciplinary operative approach, facilitating preservation of vascular integrity and functional recovery. In patients with severely displaced proximal humerus fracture-dislocations, particularly when neurovascular injury is suspected, early CT angiography should be strongly considered to guide surgical decision-making and optimise outcomes.

## Introduction

Fracture-dislocations of the proximal humerus represent a relatively small subset of shoulder injuries, yet they carry a disproportionately high risk of complications when compared with isolated fractures or dislocations [1-3]. Among these, neurovascular injury, though rare, remains one of the most serious, with the potential to cause permanent functional impairment or even threaten limb viability if not recognised promptly [1,2]. The axillary artery and brachial plexus are particularly vulnerable due to their close anatomical relationship with the humeral head, and injury may occur through mechanisms such as direct compression, traction, or laceration [3,4].

Diagnosis of associated vascular injury can be challenging, as preserved distal pulses may mask underlying vascular injury, particularly when collateral circulation remains intact [2,4]. Consequently, high clinical suspicion and timely imaging are paramount. Advanced imaging modalities, particularly CT angiography, have become indispensable in recent years, providing a detailed assessment of both the fracture pattern and its relationship to neurovascular structures [4-6]. Early and accurate diagnosis not only facilitates surgical planning but also minimises the risk of delayed treatment, which has been associated with poorer outcomes [5].

This case report describes an uncommon presentation of proximal humerus fracture-dislocation complicated by axillary artery compression and brachial plexus tenting. It highlights the critical role of CT angiography in confirming the diagnosis, guiding operative strategy, and ultimately preserving limb viability.

## Case presentation

**Figure 1:**
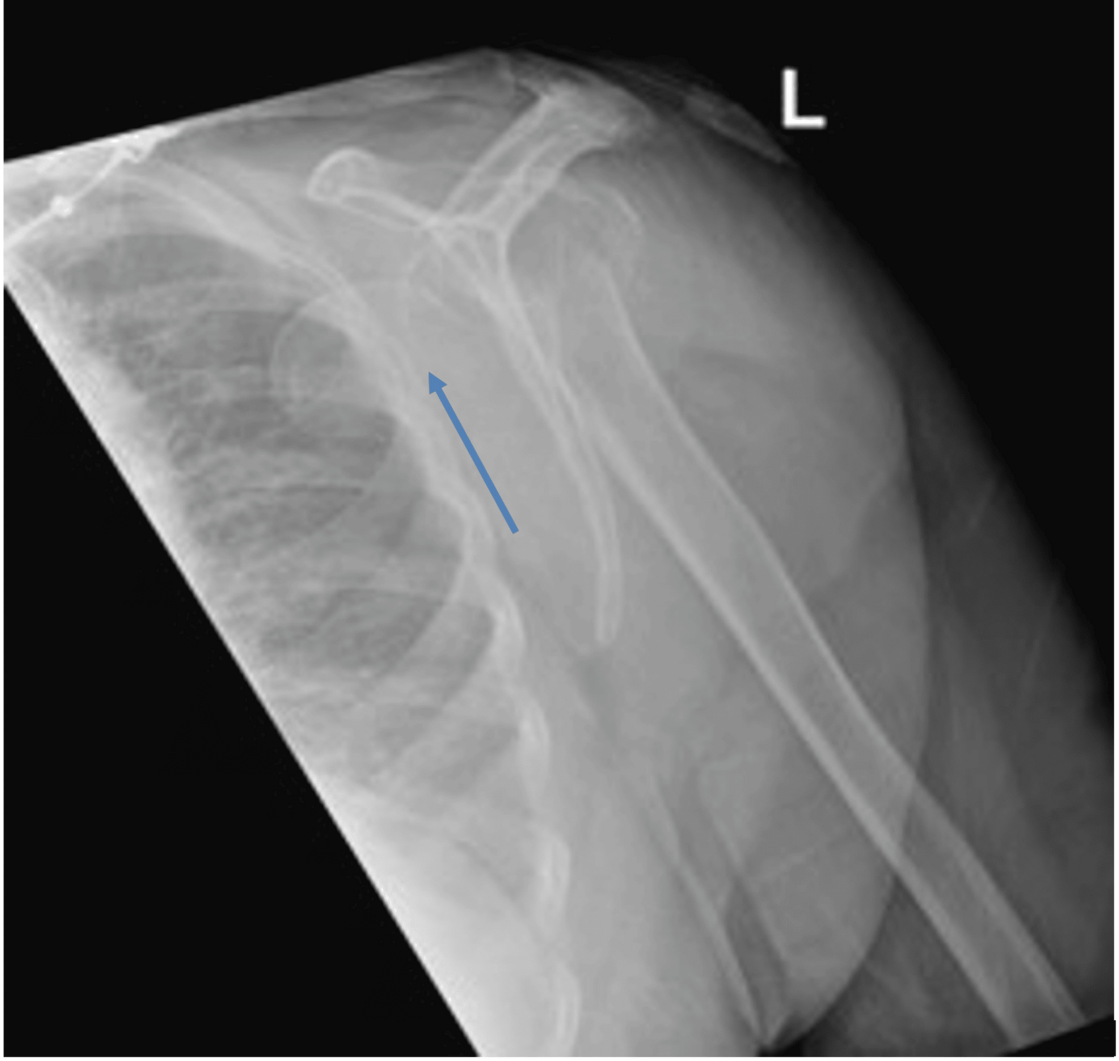
Lateral radiograph showing medial displacement of the humeral head deep into the axilla. The arrow depicts the displaced humeral head.

**Figure 2:**
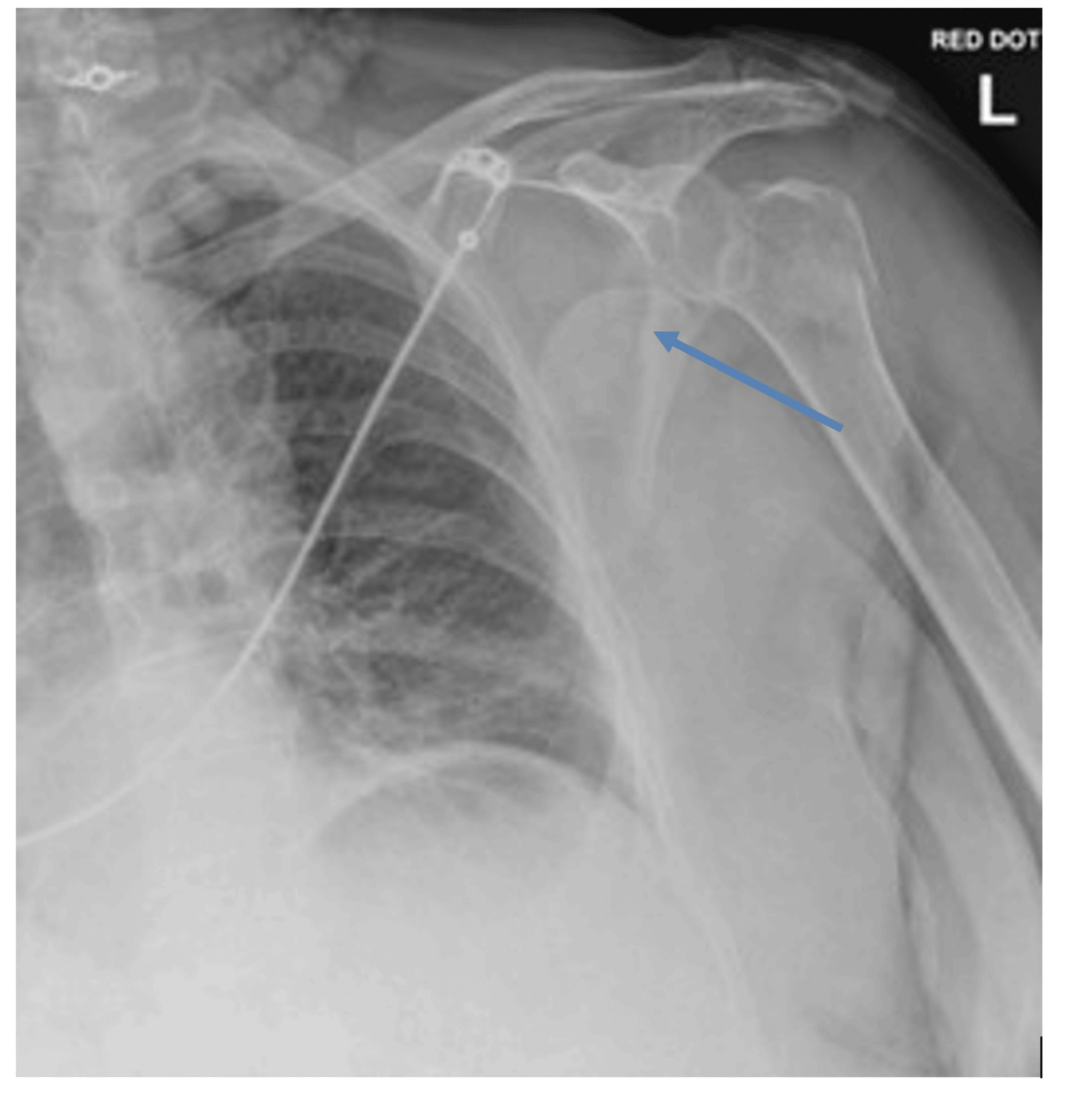
Anteroposterior radiograph showing a comminuted fracture-dislocation of the proximal humerus. The arrow depicts the displaced humeral head.

A 79-year-old right-handed female with a background of osteoporosis presented to the emergency department after a mechanical fall from standing height. She reported severe left shoulder pain and an inability to move the arm. On examination, the left upper limb was held in adduction with a limited active or passive range of motion. Distal pulses were palpable, but neurologic assessment was limited due to pain. Plain radiographs of the shoulder revealed a comminuted fracture-dislocation of the proximal humerus (Figures 1, 2), similar to those described by Robinson et al., which can predispose patients to both vascular and neural compromise [1].

**Figure 3:**
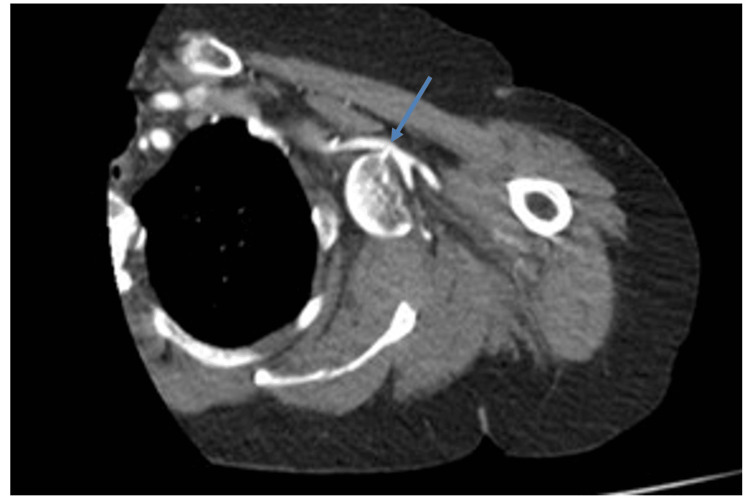
Axial CT angiogram showing the humeral head displaced into the axilla with close proximity to the axillary artery. The arrow is depicting the point of contact between the humeral head and axillary artery.

To assess for possible neurovascular injury, a CT angiogram of the upper limb was performed. This revealed, as can be demonstrated in Figure 3, the humeral head displaced medially into the axilla. In addition, a bony spike from the fractured humerus abuts the axillary artery, with no active extravasation but close contact raising concerns for compression or intimal injury. It also demonstrated the tenting of the musculocutaneous nerve, visualised as displacement and stretching over the medial aspect of the displaced head. Given the concern for neurovascular compromise, the patient underwent urgent surgical management.

Surgical management

A reverse total shoulder arthroplasty was performed for the management of the proximal humerus fracture. Intraoperatively, the conjoint tendon was taken down to enable adequate exposure of the brachial plexus. The humeral head was carefully removed from the axilla. There was no arterial rupture, but close contact between bone and artery was observed, thus confirming imaging findings. The musculocutaneous nerve was intact but visibly stretched. Postoperatively, the patient developed signs of a left brachial plexus neuropraxia, presenting with ongoing neuropathic pain and motor deficits. Clinical examination revealed the following: (1) axillary nerve: sensation present but altered; deltoid muscle strength graded at 2-3/5; (2) radial nerve: altered sensation; complete motor deficit (0/5) with wrist drop; (3) musculocutaneous nerve: altered sensation in the distribution of the lateral cutaneous nerve of the forearm; elbow flexion was difficult to assess accurately but appeared absent. It remained unclear whether these findings were pre-existing or developed postoperatively; (4) median nerve: normal sensation; abductor pollicis brevis (APB) power 3/5; and (5) ulnar nerve: normal sensation; interossei power 2-3/5. These findings were consistent with a neuropraxia affecting multiple cords of the brachial plexus. Management included application of a Futura wrist splint and referral to hand therapy and physiotherapy for neuro-rehabilitation. At the latest follow-up, gradual motor and sensory recovery was observed, and pain was improving.

## Discussion

This case highlights an uncommon but clinically significant complication of proximal humerus fracture-dislocations: axillary artery compression with concurrent brachial plexus tenting. Clinical examination alone may fail to reveal vascular compromise, particularly when collateral circulation preserves distal perfusion [2,3,5]. In our patient, the extent of humeral head displacement into the axilla was apparent on plain radiographs (Figures 1, 2), while CT angiography (Figure 3) precisely delineated its spatial relationship to the axillary artery, informing the need for meticulous preoperative planning and potential brachial plexus exploration [4,5].

CT angiography proved crucial in guiding surgical strategy, consistent with previous reports emphasising its value in complex proximal humerus fractures when neurovascular compromise is suspected [4,6,7]. While older literature relied on conventional arteriography or intraoperative findings, these approaches may delay recognition and intervention [3,5]. Intraoperatively, close contact between the humeral head and the axillary artery, along with tenting of the musculocutaneous nerve, confirmed the radiological assessment. No neurovascular structures were transected; however, the patient developed postoperative neuropraxia involving predominantly the posterior and lateral cords, affecting the radial, musculocutaneous, and axillary nerves. Nerve injuries associated with humeral fractures have been described in prior literature, but these generally arise from traction or displacement rather than direct bone-induced tenting, highlighting the unusual mechanism in our case [7]. The injury mechanism was likely multifactorial, combining preoperative compression from the displaced humeral head, intraoperative manipulation, and traction forces, which echoes findings in other high-energy shoulder trauma series [3,6,8].

Furthermore, a systematic review published in 2024 examined proximal humerus fractures with associated vascular injuries, noting that while these injuries are rare, they are often missed in individuals presenting with minor blunt trauma. The review emphasised the importance of early diagnosis and treatment, as delays can lead to increased morbidity and mortality [6]. In addition to vascular concerns, brachial plexus injuries remain a significant complication in proximal humerus fractures. A 2023 case report described a 75-year-old female who sustained a fracture-dislocation of the proximal humerus with associated brachial plexus injury [7]. Despite initial neurological deficits, the patient showed significant improvement following closed reduction and physiotherapy, highlighting the potential for recovery even in severe cases [7].

These recent findings align with earlier studies by Robinson et al. and McLaughlin et al., who also noted the challenges and complexities associated with managing neurovascular injuries in proximal humerus fractures. The integration of advanced imaging techniques, such as CT angiography, has been pivotal in identifying vascular injuries and planning appropriate surgical interventions [1,2].

Compared with the existing literature, this case underscores the importance of preoperative imaging not only for detecting overt vascular injuries but also for anticipating mechanical nerve tenting by bone fragments. The combination of plain radiographs and CT angiography allowed for a multidisciplinary surgical approach, which likely contributed to the preservation of vascular integrity despite postoperative neuropraxia. These findings support a broader recommendation for early CT angiographic assessment in patients with severely displaced proximal humerus fractures to guide surgical planning and optimise outcomes [1-8].

Learning points

Always consider CT angiography in displaced proximal humerus fractures with suspected neurovascular compromise. Bone spikes may cause compression or nerve tenting even in the absence of overt vascular injury. Multidisciplinary surgical planning, including plexus exploration and vascular involvement, is advisable in complex fracture-dislocations.

## Conclusions

Proximal humerus fracture-dislocations with concurrent vascular and neurological compromise are rare and can present with subtle clinical signs despite significant underlying injury. This case supports the role of early advanced imaging, particularly CT angiography, in patients with severely displaced fractures. Such imaging enables precise anatomical assessment, timely multidisciplinary intervention, and the optimisation of vascular preservation.
